# Gendered constructions of the impact of HIV and AIDS in the context of the HIV-positive seroconcordant heterosexual relationship

**DOI:** 10.7448/IAS.16.1.18021

**Published:** 2013-05-15

**Authors:** Anil Bhagwanjee, Kaymarlin Govender, Candice Reardon, Leigh Johnstone, Gavin George, Sarah Gordon

**Affiliations:** 1 School of Psychology (Howard College), University of KwaZulu-Natal, P. Bag X54001, Durban 4000, South Africa; 2 Health Economics and HIV/AIDS Research Division (HEARD), University of KwaZulu-Natal, P. Bag X54001, Durban 4000, South Africa

**Keywords:** hegemonic masculinities, gendered identities, South Africa, mining

## Abstract

**Introduction:**

This article explores the complex, dynamic and contextual frameworks within which men working in a mining community and their live-in long-term partners or spouses (termed “couples” in this study) respond to the introduction of HIV into their heterosexual relationships; the way in which partners adopt gendered positions in enabling them to make sense of their illness; how they negotiate their respective masculine and feminine roles in response to the need for HIV-related lifestyle changes; as well as the gendered nature of partner support in relation to antiretroviral therapy (ARV) adherence.

**Methods:**

We conducted an in-depth qualitative study with a sample of 12 HIV-positive seroconcordant heterosexual couples in a South African mining organization. Transcripts based on semi-structured couple's interviews were analyzed using an inductive emergent thematic analytical method.

**Results:**

The findings present compelling evidence that the impact of HIV and AIDS is mitigated, in the main, by the nature of the dyadic relationship. Where power and agency were skewed in accordance with traditional gender scripts, the impact of HIV and AIDS was deleterious in terms of negotiating disclosure, meeting expectations of care and support, and promoting treatment adherence. As a corollary, the study also revealed that where the relational dynamic evidenced a more equitable distribution of power, the challenge of negotiating illness was embraced in a way that strengthened the couples’ affiliation in profound ways, manifested not simply in a reduction in risk behaviours, but in both partner's courage to re-visit sensitive issues related to managing their relationship in the context of a debilitating illness.

**Conclusions:**

Gendered positioning (by self and others) was found to play a crucial role in the way couples experienced HIV and ARV treatment, and underscored the positive role of a couples-counselling approach in the negotiation of the illness experience. However, as part of a broader social project, the findings highlight the need to address the shortcomings of a public health discourse on illness normalization that reifies and reinforces skewed gender relations. In essence, the findings make a compelling case for targeting couples as the primary unit of analysis and intervention in HIV and AIDS praxis, not only to enhance treatment and prevention outcomes, but to impact on and potentially transform the lived identity of such relationships, in AIDS-affected communities. We recommend early intervention with couples in terms of couples HIV testing, risk-reduction counselling and gender-based interventions giving couples opportunities to revisit and challenge their prevailing gendered identities. We note, however, that these efforts will be undermined in the long term, if the structural drivers of HIV risk and vulnerability, contained within macro-level social, economic and cultural practices, are not simultaneously addressed.

## Introduction

Two-thirds of the people living with HIV and AIDS worldwide reside in Sub-Saharan Africa [1]. South Africa is home to the largest population of people living with HIV/AIDS, with an estimated 5.38 million HIV-positive people in 2011 [2]. Studies show that unprotected heterosexual intercourse within the context of a committed relationship is a primary source of HIV infection [3–7], with unprotected heterosexual sex being the epidemic's driving force in Sub-Saharan Africa [1], particularly amongst mine workers in South Africa [8,9]. Research has shown that mineworkers have the highest HIV prevalence rate in comparison to other sectors in South Africa [10]. HIV prevalence figures as high as 24 and 29% have been found amongst mineworkers at Anglo-Platinum and within the Carlton gold mining community [11,12]. In 2006, gold mines recorded the highest HIV infection figures in South Africa (29.2%) followed by platinum mines (20.3%), coal mines (17.6%) and diamond mines (10.2%) [13].

The mining industry employs a work force composed largely of male migrant labourers [10,14]. Male employees generally relocate so that they are closer to their source of employment, and return home after extensive periods of time [8], thereby resulting in a lack of social and family support. As a result of being away from their family and partners, and an increased exposure to commercial sex workers, migrant labourers are inclined to engage in casual sex and/or utilize the services of commercial sex workers, become infected during their stay away from home, and run the risk of returning home with HIV infection, albeit often unaware of their altered HIV status [8]. Lurie *et al*. [14] noted that amongst their sample, the main risk factors for male HIV infection included being a migrant labourer, having lived in four or more places during a lifetime, and having ever used a condom.

USAID [15] identified the recruitment of heterosexual couples as a primary target for HIV and AIDS-related research and intervention, with the couple emerging as a focal point in the HIV prevention, education, testing and treatment continuum. In particular, there is a need to conceptualize how men and women living with HIV and AIDS understand themselves and their identities in relation to their illness, integrate support into their lives and negotiate the impacts of this condition on their psychosocial well-being [16]. Given the high prevalence of HIV infection amongst mineworkers and the relatively high levels of risk that they are exposed to in terms of HIV infection, heterosexual male mineworkers and their spouses or long-term live-in partners, who were HIV positive and seroconcordant, were selected as the sample of couples for this study.

### Gender as a relational construct

In this paper, we adopt a socially constructed theory of gender because “gender” is pertinent in relation to how people view themselves and their partners within the context of their heterosexual relationships. Constructionist theories regard gender as something that people achieve rather than something they are born with; it is a social accomplishment dependent on gender-related structural and symbolic processes that underpin social interactions [17] and invariably constitute a gendered cultural discourse [18,19].

In the relational context, social interaction can be interpreted according to how individuals position themselves or are positioned by others. Positioning theory posits that “subject positions” are situated within the context of power dynamics, which reflect moral or personal attributes that correspond to a particular “right” or “duty” [20]. For instance, “a person who is positioned as “powerful” might be assigned the right to inflict violence, whereas a person who is positioned as “powerless” might be assigned the duty to receive the violence without resistance” [21, p. 248]. The way in which people are positioned, and position themselves, in terms of the assignment of rights and duties, thus facilitates how they act within a given social context [22].

Against this backdrop, gendered narratives and gender itself can be seen, in part, as simultaneously the producer as well as the product of power. Pyke [23] accordingly takes the view that societal structures of power and inequality are substantiated through the micro-level practices involved in structuring the social relationships of everyday life, which are in turn constituted in the practice of health behaviour, amongst other practices. Gender inequalities are enabled through gendered constructions of health and health behaviour [24]. In this way, men may use hegemonic masculine health beliefs and practices as a way of establishing themselves as men; for example, by denying weakness or vulnerability, suppressing their emotional and physical crises and refusing to acknowledge or admit their pain [24]. For women, in contrast, expressing their emotionality and acknowledging their vulnerability in the face of illness is usually associated with help seeking behaviours [25]. Building on this literature, we accordingly conceptualize gender as the institutional, relational and identity-related conceptions of selves operating within particular situational contexts.

### Hegemonic masculinities, femininities and HIV and AIDS

Connell [26] argues that many societies subscribe to a “dominant” (hegemonic) definition of masculinity, which both reifies and authenticates male power while subordinating women and other non-dominant forms of masculinity. In this regard, Brown *et al*. [27] assert that cultural groups construct “ideal notions of masculinity”, which bring into existence the hegemonic masculinity - the masculinity “that men measure themselves against, and are measured against by others” [20, p. 586]. This hegemonic masculinity thus dictates and values certain beliefs, behaviours and desires in men, thereby subjugating both undesirable masculine traits and femininities as its opposite and “other” [21].

There is a growing body of literature which places increased recognition on how hegemonic forms of masculinity serve to reinforce gender inequalities and in so doing places both men and women at higher risk of contracting HIV [28–33]. This literature identifies hegemonic notions of a “real man” (viz. hard, self-determining, physically strong, fearless and sexually inexorable) as key drivers of the AIDS epidemic in many African contexts [34], where the development of hegemonic masculinity has been shaped by lived socio-cultural and political histories. In South Africa, for instance, colonialism and apartheid were ideologically predicated on the destruction of the cultural and economic fabric of the indigenous peoples, such that traditional symbols of manhood, for instance land ownership and being the head of a household, were destroyed [35]. Thus, Ratele [36] describes a historically emergent “ruling” masculinity in South Africa that is constituted of “assertive heterosexuality, control of economic decisions within (and outside) the home, political authority, cultural ascendancy and support for male promiscuity” [36, p. 51]. For black African men, the material challenges of life have always been a dominant issue. Menial, poor paying jobs with no scope of professional advancement has increased the likelihood of finding masculine affirmation in homosocial (sometimes criminal) settings and their (subordinated) relationships with black women [37]. In the context of HIV and AIDS, it is argued that these hegemonic masculinities promote multiple sexual partnerships, reduce male access to health services as well as reduce the likelihood of males adopting safe-sex practices [30,38].

As a corollary, discourses of women cannot be viewed outside the realm of male sexuality. In this regard, feminist work has highlighted the repression of women's sexual agency as a significant aspect of gender power inequality within a discourse of heterosexuality [39,40]. Previous research in South Africa has recognized the high levels of violence against women and coercion in sexual relationships as critical drivers of vulnerability to HIV infection [41,42]. In fact, women with violent and controlling partners have been shown both to have more frequent sex and to use condoms less often [43], endure physical punishment [44] and face the threat of abandonment [44] if they do not “give in” to male pressure for sex because of “love” and commitment [45]. Jewkes and Morrell [37] assert that the dominant form of femininity thus requires “women to be strong, and able to accept and cope with the stresses life brings, including those caused by women's subordinate position in their relationships” (p. 6).

These broad systems of inequality are not uni-dimensional, in that they can simultaneously contain and produce counter-discourses that operate as oppositional and productive forces against gendered power relations, particularly in the context of HIV. Drawing on Foucault's [46] understanding of power, these counter-discourses are tangible and can surpass deeply entrenched gender inequality. For example, Morrell's conception [47] of progressive masculinities in South Africa, that upholds gender equality and the right to homosexual practises, and which seeks to promote new versions of manhood that are non-violent, counter the norms of traditional masculinities. In a similar vein, Mfecane [48] describes the “transformed masculinity” demonstrated by South African men on antiretroviral therapy (ARV), who reconstructed their masculinity in order to avoid harmful HIV-related practices associated with hegemonic masculinity, such as multiple sexual partnerships.

With respect to women's accounts, Shefer and Foster [49], as well as Reddy and Dunne [50], provide evidence of marginalized counter discourses that challenge hegemonic constructions of women as passive, lacking in sexual desire and simplistically responsive to male active sexuality. However, outside urban South African settings, where the “modern girl” femininities [37] are able to exercise a significant measure of independence, most women, especially black African women, are generally economically dependent on men and constrained within discourses of cultural obedience. Therefore, while progressive counter-discourses in women's accounts of heterosexuality in the South African context can serve to increase their personal agency, the evidence does, in the main, highlight the fragility and ambiguity in the processes of identity construction in women in a high HIV prevalence context.

Drawing on research undertaken with a sample of HIV-positive seroconcordant heterosexual couples in a mining sector workplace in South Africa, this study explored how men and their long-term live-in women partners or spouses make sense of their emergent realities both at an individual and collective level [51,52], and the implications for identity, meaningfulness and a sense of belonging in relation to their illness and specifically ARV adherence. This study was concerned with the following specific research questions:

- How do gendered positions manifest in the relationships of couples where both partners are infected with HIV?
- How do these couples, in the context of existing gender inequalities, negotiate their respective masculine and feminine identities in response to the need for agency in making HIV-related lifestyle changes?
- What is the impact of gender and relational dynamics on partner support for ARV adherence?

## Methods

### The study site

This study was part of a broader research project on HIV and AIDS conducted in the mining sector and was based at one of the sites of a multinational mining company located in South Africa. The mining company that was chosen for the study had institutionalized a comprehensive HIV and AIDS prevention, testing and treatment programme over the previous eight years. Free HIV testing and treatment was available to all permanent employees and contractors, as well as to their spouses/life partners, and was managed by an on-site company clinic, which was staffed by a doctor and two full-time nurses. The clinic provided comprehensive medical services, from routine medical screening through to treatment for chronic clinical conditions, the costs of which were borne by the company and a closed medical aid scheme.

### Sample and sampling demographics

At the time of the study, 104 HIV seropositive patients had historically been registered on the clinic's treatment programme. Some of these patients had recently been retrenched, but in terms of the company HIV and AIDS policy, still enjoyed access to free clinical services, including treatment for HIV and AIDS. Of the total patient cohort, 38 individuals were on HAART (Highly Active Antiretroviral Therapy) and 40 on pre-HAART, with the balance being deceased, or out of contact with the clinic. HIV-positive employees on treatment were encouraged to bring their spouses along for HIV testing, counselling and treatment, the costs of which were also borne by the company and the closed medical aid scheme.

Sixteen couples had been identified as being HIV positive, were on the clinic's treatment programme and were fluent in English. All 16 couples that satisfied these inclusion criteria were invited by the company medical practitioner to participate in the study; 13 couples agreed and accordingly gave their informed consent to participate, but one couple had to withdraw on the day of the interview due to illness of one partner. Of the remaining three couples, one couple turned down the doctor's invitation for unspecified reasons and the other two were unavailable on the days assigned for fieldwork because of family and/or personal commitments. No couple refused to participate on the grounds of interviewing on a topic that might be offensive or sensitive.

The partners had been together for between 5 and 15 years at the time of the study. The majority of participants had been aware of their HIV status for four to five years, one male participant had known about his status for eight years and one female participant had known about her status for one year. Fifteen of the 24 participants were currently taking ARVs and one participant had stopped her ARV treatment two years ago. Eight of the couples were married and four were in long-term partnerships.

### Data collection and procedure

Semi-structured interviews were conducted with each couple between April and June 2010. Participation was emphasized as voluntary and participants were offered a small stipend for participating in the study. Fieldwork was conducted at the clinic by a research team that was independent of the company and the clinic staff. Interviews were conducted primarily in English, and where necessary, the interviewer was able to switch to Afrikaans or Setswana (the local languages of the participants) for purposes of clarification. Standard protocols were followed in securing ethical approval for the study from the University of KwaZulu-Natal.

In depth, semi-structured couples interviews were conducted because this method is designed to detect, represent and elucidate the meanings associated with particular viewpoints from the individual actors as part of a relational dynamic [53]. Emphasis was placed on understanding how each partner in the dyad made sense of their experiences, the meanings they derived from their own experiences and the manner in which they related to each other. Having both partners present allowed each to represent their issues, claims and concerns in a safe and structured space. Giving a voice to participants’ own representations of a shared reality, in the presence of the partner, is important as it acknowledges their agency [54] in terms of how they negotiated their respective masculine and feminine identities in response to the need for HIV-related lifestyle changes, including partner support for ARV adherence [55].

Given the sensitive nature of the research area and the potential risks associated with interviewing couples together, special care was taken in ensuring that a suitably qualified and experienced clinical psychologist conducted the research, such that relational issues were privileged and potential conflict and psychodynamics were appropriately managed. Upon completion of the interview, the interviewer, with the informed consent of the participants, provided the clinic medical practitioner with feedback pertaining only to issues requiring referral and/or follow-up (four such referrals were made: two for couples counselling and two related to specific difficulties in adherence with ARVs).

### Data analysis

Interviews were initiated, digitally recorded, transcribed verbatim and examined through a set of systematic procedures [56]. Specifically, a thematic analysis of interview data was conducted using aspects of Ritchie and Spencer's [57] framework approach and Neuman's [58] inductive emergent approach. Transcripts of the interviews were coded and the data analyzed for patterns of consensus, contrast and variability. Transcripts of each interview were then analyzed line-by-line and codes were developed to label key themes in the data. Codes were generated inductively, using open coding methodology, in which information was broken down, conceptualized and categorized. Finally, themes were derived that reflected the emergent nature of the analysis. Field notes and fieldworker observations were used as additional data sources to validate and elaborate the thematic findings.

## Results and discussion

### Gendered positionings of HIV in the couple's relationship

#### Discourses of illness normalization and the role of ARV adherence

The discourse of normalization positions people living with HIV and AIDS as chronic sufferers and invokes the need to manage their condition by adhering to their ARV medication, exercising, eating healthily and managing stress and anxiety. These constructions reflect an important public health discourse that is critical to the establishment of agency in dealing with illness [59].

On one level, it was apparent that several participants had positioned HIV as an illness that had to be managed. In the following quote, a male participant articulates this view:

But you must treat it like any other disease, otherwise you will hurt yourself. As long as you eat well, exercise and take your medication, you will live long, in fact longer than people who are HIV negative.

In the face of the very real public health threat presented by HIV and AIDS, the above discourse is critical in offering containment, reduction of fear and the enablement of a range of healthy behaviours and lifestyle changes [60]. For instance, in a study on HIV-positive male mine-workers in South Africa, Bhagwanjee *et al*. [61] found that participants associated their control over their adherence behaviours with the belief that that they could live a long, healthy and normal life despite their HIV infection.

Further, participants’ views on adherence appeared to be reinforced by a positive relationship with the clinic's doctor, as demonstrated in the following excerpt from a male participant:

Last time we visited the doctor he told me that I look great. He constantly gives us feedback on how we are doing and is always honest with us. This is really helpful when you go off course. He counsels us when we slip up and now I can tell you that nothing will stop us both from sticking to our treatment. We have been with our doctor for many years, and now we know we can do it.

In general, it was noted that the positive relationship couples shared with their doctor, and specifically the support and guidance that they have enjoyed, helped establish agency and consequently normalization of the illness. The positive impact of the health care provider is echoed in previous research; for instance, Johnson *et al*. [62] demonstrated how positive patient-provider interactions fundamentally influenced both adherence self-efficacy as well as adherence behaviour.

#### Living with HIV: vulnerability and invincibility

Notwithstanding this normalization discourse, particular gendered constructions and positionings of HIV emerged in the interviews that ran counter to notions of adjustment and acceptance. At regular points in the interviews, many of the females described living with HIV as a painful, challenging, confusing experience and were open about the emotional struggles they were engaging with at the time of the interview, while the male perspective with regard to living with HIV attested to a perceived sense of invulnerability and indifference in the face of others:

Interviewer: What does it mean for you to live with HIIV?

Male participant: I don't care anymore about what people say. They have been counting the nails in my coffin, but I am still here.

Interviewer: What does living with HIV mean to you, Mrs. Jane [not real name]?

Female participant: It is very painful. People treat you like you have some kind of poison, and that you are going to infect them. You also get other diseases in the process, which makes it very worrying day-by-day.

In another couple's interview, the female partner demonstrated her vulnerability deriving from her concerns around childcare and nurturance, while her husband positioned and responded to the threat of HIV in a seemingly rational and emotionally distant way:

Interviewer: “What does being HIV positive mean to you?

Male participant: We are sick and we need to take care of ourselves-we can overcome this thing.

Female participant: I wish that I wouldn't die soon because I want to be here to look after my kids as they grow up.”

The above excerpts suggest that the normalization discourse allows little space for the integration of many of the women's emotional and relational struggles. Moreover, they illustrate the male hegemonic position of invulnerability in the face of the seriousness of the illness, which is self-perpetuating. These findings are echoed in Skovdal *et al*.'s [63] study, which illustrated that when men internalized HIV and AIDS, the illness represented a threat to their manhood and dignity. By distancing themselves from any association with the emotional weight of the illness (as demonstrated in the above two quotes), male participants were able to reproduce and maintain their masculine power. Such forms of denial, however, place men in a vulnerable position as it may lead to a failure to access medical or other forms of support [34], including HIV testing and treatment services [64]. It also serves to distance men from acknowledging the very real emotional concerns of their partners, and in so doing, acts to reinforce the hegemonic masculinity that Courtenay [24] is concerned with.

### The reinforcement and strengthening of gender roles and inequalities in couples’ relationships

#### Disclosure, betrayal, helplessness and victimology

Disclosure of HIV status by males left the majority of female partners feeling shocked, confused, angry and even suicidal, particularly because of the perceived unfaithfulness of their partners, their partners’ deceit in concealing their HIV status and the possibility that their partner probably infected them with HIV. In the following excerpt, a female participant describes how difficult it was to accept that she was infected with HIV in spite of being faithful to her husband:

Female participant: “I was hurting because I could not understand why I got it (HIV) because I stay at home, I don't have friends and I don't go anywhere”.

Interviewer: You are saying that you were faithful to him, always?

Female participant: I was always faithful. I always stayed at home with the children waiting for him to come back from work. So, I can't understand, how did I get it from staying at home? (She cries; husband looks down quietly).

Several female participants reported experiencing much worry and concern about their husband's deteriorating health and risky sexual behaviour, and felt powerless to intervene in transforming their conditions of living. In the following excerpt, one of the women expresses this concern:

Female participant: He's still naughty. It is not good …. He is re-infecting himself. Talk to him, he is naughty.

Interviewer: How does it make you feel then?

Female participant: I don't talk too much. I just look at him just like that. He does not want to listen.

Interviewer: How did it make you feel when the counsellor says he is HIV positive and you have been telling him to behave properly?

Female participant: It hurt me because he just does not want to listen … he is hurting himself by continuing with this behaviour.

Both female participants quoted above evidenced hurt and pain associated with betrayal and both did so with a sense of powerlessness and desperation that relegated them to the victim/spectator position [20]. This sense of helplessness associated with the victim role is as much a mirror to the gendered power differentials at play in men and women's negotiation of disclosure and betrayal, as it is a reflection of the co-option of women in reinforcing gendered constructions of victimology [5], which are predictive of negative relational and health outcomes for both partners [29,30].

#### Living with the other: acquiescence and agency

Despite the physical and emotional challenges in their relationships, the majority of female participants reported feeling compelled to remain with and care for their partners despite being infected by them. In the following quote, one female participant describes this obligation to care for her husband. It is also interesting to note that she is very reluctant to impose her care needs on her children:

Female participant: I don't like the way he talks to me sometimes. I always think about leaving him, but I would not take an already rotten thing to my children's home. He is the one who is supposed to take care of me. And the way he is, I cannot leave him like this … yet no one does anything for me even when I am sick.

Interviewer: What did you mean about the rotten thing?

Female participant: I mean someone who is diseased and helpless. Sometimes it becomes difficult for people to take care of you, when you are like that.

The above results reveal that the entry of HIV into the couple's relationship served to reinforce gender roles. Women's sense of their “spousal duty” meant that they were being positioned, and positioned themselves [20] as the primary carer and supporter of their husbands (and children) despite the anger, pain and betrayal which they so palpably evidenced, and despite not receiving reciprocal care. The extract above serves as an illustration of the micro-level practices’ that Pyke [23] refers to, which serve to promote and reconstruct broader systems of inequality.

Similarly, after learning about her husband's infidelity and his HIV status through discovering his ARV medication at home, one female participant was forbidden by the elders in her family to leave her husband, notwithstanding the fact that he was probably the one who had infected her. Despite her husband concealing his HIV status from her, his alcohol abuse and continued infidelity, she articulates her responsibility as a carer and loyal wife:

You know how we black people are, when you are married you need to take care of your husband regardless of what he is doing to you … I was married traditionally and I also went to church so I'm not going to leave him and become a burden to other people who will be suffering already because that is not going to change anything. I'm HIV positive and divorcing him won't help me or change my status. Whoever becomes ill before the other must take care of the other. I'm not going anywhere.

Both participants above illustrate their unwavering commitment to traditional cultural norms. In the African tradition of lobola, the symbolic “purchase” of the bride embodies this commitment. The excerpts point to the complex and mutually reinforcing interplay between gendered identities and cultural and social systems that serve to mutually reinforce dominant forms of masculinity in South Africa [36].

In terms of positioning theory, power and agency are dependent on how people are positioned (and/or position themselves) through the assignment of rights and duties [20]. Within this context, women in this study have largely positioned themselves with the “duty” of being a carer for the entire family, thereby reducing their own agency in terms of the (re)assignment of this role. In addition, women provided support to their husbands, even at their own physical and emotional expense, thereby facilitating the maintenance of their husbands’ hegemonic behaviours (and traditional masculine position), despite being subordinated by them. In fact, Bhagwanjee *et al*. [61] reported that despite the support rendered by their female partners, most of the men in their study continued to remain unfaithful, thereby actively maintaining their hegemonic masculine position.

It is important to note, however, that despite this apparently subjugated gendered identity, both female participants also demonstrated a defiant and unwavering resolve to remain in the relationship and make it work. This sense of autonomy and agency underscores the fluid and dialectical nature of lived gendered identity, and speaks to the marginalized counter discourses that Shefer and Foster [49] invoke, and that might exist side-by-side in relational space to a hegemonic construction of women as passive and reactive to male power and sexuality.

### Challenging hegemonic roles and power in the couples’ relationships

#### Non-hegemonic masculinities and counter discourses of femininity

In the majority of the couples’ narratives it was evident that the male partner held the dominant position in their relationship. However, in the case of two couples, we noted that the male partners were attempting to adopt a less traditional, more egalitarian relational position. The male participant in the following quote indicated that he had become conscious of the negative impact of his behaviour on his family and that he did not want to cause them further undue stress:

Male participant: I don't like to make her angry …. I used to drink a lot before so I reduced my rate of drinking because I noticed that alcohol will destroy my family. I would come back home drunk and that usually caused chaos in my family. So I don't want to bother my wife anymore because I know her state.

In another couple's relationship, the husband had similarly indicated that he had stopped having sex with multiple partners after finding out he was HIV positive and had asked his partner, whom he had infected with HIV, to marry him. In this interview, both partners talked about engaging in joint decision-making around who they were going to disclose this information to. The following excerpt from their interview illustrates how they co-constructed the notion of a healthy lifestyle and the extent to which they depended on each other for emotional strength and support during this process:

Male participant: I think she is much stronger than I am. She has helped me a lot in the whole process.

Female participant: Since that day, we have decided to live a healthy life. We decided that there will be no more alcohol and smoking. We decided to buy healthy food.

Interviewer: You said she is stronger than you i.e. if one is to compare the two of you?

Male participant: Yes.

Interviewer: What do you mean by that?

Male participant: She has helped me a lot through this process. Even after she was diagnosed, she handled it better than I did. But when she went for her CD4 cell count she cried because I was not there.

The above extracts vindicates Foucault's [46] arguments related to the productive function of power, where the female partners were able to exercise agency in helping their male partners to shift their masculinities in a reciprocal and mutually reinforcing way. In practicing fidelity, not engaging in smoking and alcohol abuse, shifting to a healthy diet and in co-creating the relationship (and especially the admission by the second male participant that his partner is stronger than him), these two male participants demonstrate elements of a more “progressive masculinity” [47]. Importantly, the relational shift described by the two couples appeared to have become possible as a result of a counter discourse from the female partners that challenged traditional constructions of womanhood [50].

#### Female agency in sexual interactions

Within the traditional have/hold discourse, condoms are often discarded when a couple enters marriage or a committed relationship [3,65]. Accordingly, women are often unable to insist on continuous condom-use in their long-term relationships as this may be construed as a sign of unfaithfulness or disobedience on their part, which could instigate abuse [66]. This is often compounded by gender inequalities in relationships, which compromise a woman's agency in negotiating the use of condoms in particular circumstances [5,66,67].

In spite of entrenched traditional gendered relations in other domains of the relationship, 10 out of the 12 female partners in this study reported that they had taken control of sexual decision-making in their relationships after both partners had disclosed their HIV status. In particular, female partners reported that they decided when they were going to have sex with their partners and insisted on using a condom when they did so. In the extracts that follow, we discuss this more fully in the context of HIV-positive seroconcordant relationships.

The empowered female stance towards sexual activity was evident in women who were able to refuse the sexual advances of their husbands and boyfriends if they did not feel like it.

Interviewer: Are there any changes in the house in terms of decision-making, since you had both been diagnosed HIV positive?

Male participant: No one dictates to the other. We both come up with the suggestions and when her suggestions are better than mine, we go with it and if my suggestions are better than hers, we take mine.

Interviewer: Okay. When it comes to sexual intercourse, who makes the decisions?

Female participant: If I don't want to, I don't want to.

Male participant: That's where we fight. Because when I want it, she does not want to. Even if there are condoms, she would sometimes refuse. I'll take my blankets to the lounge and sleep there because she does not want to.

It is important to note that the husband in the above relationship was engaging in on-going infidelity, thereby providing his wife with sufficient grounds to deprive him of sex, as it was most likely this infidelity that had led to her becoming infected with HIV in the first place. The exchange above vindicates the findings from other studies which show that the introduction of safer sex in a relationship, enforced primarily by the women, invokes marital strain and tension [68]. Overall, the more empowered stance in sexual decision-making adopted by the female participants in this study demonstrates how the imperative to actively protect themselves from reinfection also resulted in a reclaiming of agency in the most intimate arena in their relationship. Chatterjee Rogers *et al*. [7] also found that the introduction of a HIV diagnosis in a relationship often leads to increased condom-use and decreased sexual activity. Further, van Devanter *et al*. [69] and van der Straten *et al*. [68] identified sexual intercourse as a significant driver of this shift in sexual relations amongst HIV-positive couples, as sex serves as a reminder of the presence of HIV in the relationship.

While in many cases, the male partners were resistant to their wife or girlfriend's insistence on condom-use and/or the decreased frequency of sex in their relationship, over half of the female participants had nevertheless managed to successfully reintroduce condoms into their relationships. In the following excerpt, one of the female partners reflects on her reasons for always using a condom with her HIV-positive partner:

Interviewer: Do you use condoms every time with your husband?

Female participant: Oh yes.

Interviewer: You sound very sure and determined about this?

Female participant: Yes, I don't want anything. The thing is we go to the clinic and I read about it so I know about re-infection. So if we keep on sleeping without a condom we will be doing damage. First before I knew about his status I used to have STIs and it did not come to my mind to do blood tests. But now it's better because we are using condoms and testing regularly and staying on treatment.

The women quoted above position themselves as being responsible for negotiating safer sex despite the dominant heterosexual discourse, which might still prevail in other aspects of the couple's life. It appears that the negotiation of sex in the dyadic relationship was seen as a viable space in which the women could *justifiably* contest their partners’ dominant position, partly due to their perception of having been infected by their partners and/or being subjected to their partner's repeated infidelity. While this discourse of responsibility required personal agency on the part of women, it also has the contradictory effect of placing the burden of care onto women to manage the couple's sexual relationship and ensure that the family is protected.

### The impact of gender and relational dynamics on partner support for ARV adherence

#### Unpacking spousal support

The majority of the sample reported sub-optimal adherence to their ARV medication, which primarily involved unintentionally missing dosages or taking them later than required. Only two participants reported intentionally stopping their ARV medications for a period of between one and two years, primarily because of negative side effects of the medication, and both were reportedly in conflict-ridden and strained relationships with their partners.

As a result of many of the couples not disclosing their HIV status to significant friends, relatives or work colleagues, the majority of the participants, as reported in other studies [70,71], depended on and looked towards their partners as their primary supporter, even if their partners did not fulfill this role satisfactorily.

The most common form of support given and received by the partners in this study was practical support in the form of physical help when the partner was weak, collecting medication for partners, reminders for treatment doses by partners, going with partners to the clinic, cooking for the partner when they were ill, and helping with household chores. It also emerged from the interviews that emotional support was rarely shared between the partners. Male partners’ support was most often conceptualized and provided in practical terms. In the following excerpt, a male participant describes the importance of partner support in helping both parties to live with a HIV-positive diagnosis:

Male participant: She was very sick. I think she won't be able to survive if I'm not there and I won't be able to survive if she is not there.

Interviewer: Mm.

Male participant: I think she is a good support system in my life and I'm a good support system in her life.

Interviewer: Do you also feel that way?

Female participant: Yes absolutely.

Many of the male partners tended to overestimate the quality of the support that they offered to their partners and the extent to which it was effectively fulfilling their partners emotional and practical support needs. For example, in the following quote a female participant voices her desire to be loved by her husband and for there to be less tension and overt conflict in their relationship:

I need him to give me strength by respecting and loving me, not just helping me with chores. I told him that I think that he does not love me anymore, because if he does we would not be fighting the way we do. It hurts me sometimes because I'm trying and he does not appreciate it … I ask that he love me and when we fight, let it be a fight that lasts for a short time only. I feel like he wants to demoralise me. Were it not for the fighting, my CD4 cell count would still have been very high.

For the majority of the female participants, a primary source of comfort was to know that their partner would remain committed and not desert them. The majority of women complained that their male partners were physically and/or emotionally distant in their relationships. The following quote illustrates how partners may have very differing views about the extent to which each other's emotional support needs are being met in their relationship:

Interviewer: Do you think the situation you are in now has affected the way you love each other?

Male participant: My love for her has not changed. I don't know about her.

Interviewer: What about you, Mrs. Janice [not real name]?

Female participant: Yes, it has. I feel like he is not there for me at all.

Interviewer: How would you like things to be?

Female participant: I want him to be there for me. But if he can't, he can't.

The unmet emotional needs of the female participants may stem from gender dynamics in how support is conceptualized, in that most of the men appeared to value practical support given to them by their partners (e.g. collecting medication, cooking, doing household chores) more than emotional support (need to be loved, respected and cared for). Perhaps due to the very poor quality of the relationship between some of the couples, emotional support was very difficult to provide, with HIV disclosure having fundamentally altered their relationships and their closeness significantly. In the following excerpt, a couple describes the type of support which they would like to receive from their partner. It is interesting to note the difference in the type of support that is desired and valued by each partner:

Interviewer: Does your partner support you?

Male participant: Yes, especially now that I am not working. She gives me some of her money.

Interviewer: Is that the kind of support you are expecting from her?

Male participant: Yes, I know that if she was working she will be giving me enough support.

Interviewer: What does support mean to you, Mrs. Cynthia [not real name]?

Female participant: It means helping each other physically and emotionally.

Interviewer: Are you happy with the support he is giving you? Is there anything else you'd like him to do?

Female participant: I want him to be there for me because money is not everything.

Male participant: Yes, she is always telling me that.

These findings are in keeping with research evidence showing that gender differences exist in the extent to which individuals provide treatment support to their partners. Eagly *et al*.'s [72] gender role theory posits that women's traditional gender roles prescribe that they provide more social support than they receive. This assertion is corroborated by empirical evidence demonstrating that women generally offer higher (and more effective) levels of support compared to men, including spousal support [73], even to partners who are ill [74]. Thus, women generally consider the giving of support in intimate relationships to be a more profound determinant of health outcomes than receiving support [16], despite the higher levels of stress that this may generate for them as compared to men [75]. In addition, women have been reported to be more likely the sole care-giver, providing, on average, double the amount of hours of care, as well as more intensive and complex care, than male caregivers [76]. In the context of this research, it should be noted that spousal support is linked to illness prevention and management, with lower levels of social support [77] and limited emotional and psychological support from others [78] being predictive of poorer levels of ARV adherence.

It is clear from the findings that relational difficulties and gender differences negatively influenced the quality and depth of partner support for ARV adherence, thereby undermining the prospects of treatment adherence for both partners. In terms of hegemonic masculinity, the notion of love becomes problematic and contested as the expression of emotions is seen as a weakness and men begin to see their bodies as a machine that they need to control [79]. To this end, perfomative and instrumental expressions of love were favoured by male partners in this study over more relational and emotional expressions and left their partners’ primary support needs largely unfulfilled, thereby perpetuating relational problems.

## Conclusions

The most striking outcome of this set of interviews is the compelling evidence showing that HIV and AIDS had a profound and devastating effect on the dyadic relationship, particularly of those couples whose relationships were already strained due to alcohol abuse, infidelity and unemployment. For 10 of the 12 couples, the entry of HIV into their relationships served to exacerbate and reproduce existing gender inequalities, generate conflict in the most intimate domain of their lives, and further polarize the partners from one another. The overall effect was the reinforcement of traditional gender scripts, with women retreating into a submissive position, despite their underlying hostility and a range of unfulfilled emotional and physical needs.

At the same time, the interviews provided evidence of the fluidity of gendered identities, with women partners resisting, and at times, challenging their subjugated gender positions. The latter was most evident in the sexual domain, which emerged as a primary site where the majority of women took a stand and challenged the traditional heterosexual script, through the adoption of a “responsible subject position”.

In this regard, our findings suggest that female agency demonstrated in the sexual domain was attributed largely to a desire to protect themselves and their partners from reinfection through consistent condom-use. By their own account, participants’ perceptions of the likelihood of re-infection by their partners arose predominantly from an emphasis on safer sex, and condom-use in particular, in education sessions provided by clinic staff. In this regard, Poudel *et al*. [80] assert that seroconcordant partners who engage in unprotected sex put themselves at risk of superinfection and/or contracting STIs. Notwithstanding the disparity in empirical findings reported with regard to the risk of superinfection faced by seroconcordant partners in the era of ART (from no superinfection to an incidence of over 18% [81]), Campell *et al*. [82] conclude that superinfection may occur despite ART and advocate that condom-use amongst HIV positive seroconcordant couples be emphasized. In their review of superinfection, Waters and Erasmus [81] conclude that the risk of transmission of resistance may be as high as the risk of new incident infection in the presence of on-going exposure. They accordingly assert that HIV-infected individuals should be counselled that there is risk of superinfection at all stages of HIV.

We concur. We argue that in the absence of substantive evidence indicating otherwise, superinfection remains a potential risk factor and must therefore remain on the health promotion agenda, with prevention, education and counselling on safer sex for couples, including in particular condom-use, being sustained [82]. Further, caution needs to be exercised not to subvert over three decades of public health messaging and patient counselling regarding condom-use on the basis of controvertible evidence, noting also the role of condoms as a primary contraceptive method in resource poor settings [83]. The issue of superinfection needs to be explored in further research, as does the potential consequences of this empowered response from female partners in the context of couple's relationships.

There was credible evidence showing that where the relational substrate was more egalitarian and more stable, the challenge of negotiating illness and its treatment was embraced in a way that strengthened the couples’ affiliation in profound ways, manifested not just in a reduction in risk behaviours, but in a manner that enabled them to muster the courage to re-visit sensitive issues related to power and agency in the context of a debilitating illness. In essence, this is the most compelling evidence for targeting couples as the primary unit of analysis and intervention in HIV and AIDS praxis, not only to enhance treatment and prevention outcomes, but to impact on and potentially transform the lived personal and social identities of couples and their families.

Our findings therefore make the case for early intervention with couples, that is, offering and encouraging couples to participate in HIV testing and risk reduction counselling prior to either partner becoming infected. The damaging influence of hegemonic masculinity on the couples’ relationships, health behaviours and support needs, as evidenced in this study, underscores the importance of on-going gender-based interventions for HIV-positive seroconcordant heterosexual couples. Such interventions should focus on providing partners with opportunities, within contained social spaces, to revisit, and, where possible, challenge prevailing gendered identities.

This research also highlights the critical role of health practitioners in positioning HIV and AIDS as a chronic condition that can be managed by adhering to ARV medication, exercising and healthy eating. However, our findings also elucidate the potential shortcomings of this normalization discourse, because it largely precludes women's narratives of pain and suffering, effectively invalidating a significant portion of their experience of living with HIV. This underscores the need to elaborate our public health discourses within a social project that transcends the bio-medical disease–treatment axis, and which, by omission at least, reifies a virus and inadvertently reinforces skewed gender relations.

However, it should be noted that HIV prevention efforts will be compromised in the long-term if the social, economic and cultural drivers of HIV risk and vulnerability are not addressed. Gupta *et al*. [84] assert that the process of implementing structural approaches must, therefore, begin with the analyses of how social, political, economic, and environmental factors operate and the pathways that lead to risk in a given community. While this study does not claim to have accomplished such an ambitious social analysis, we do provide some relevant insights in relation to how we can define and build capacity within the context of programmatic interventions with seroconcordant heterosexual couples living with HIV and AIDS, especially in mining settings, which can also translate into more sustainable community-based interventions.

Finally, the cross sectional research design and the homogeneity of the sample in terms of the length of time partners had been aware of their HIV status (between four and five years) was a limitation of the study. Future research would benefit from employing longitudinal designs to explore developmental change processes in gender identities and relationship interactions over time as HIV disclosure and acceptance unfolds within the couples relationship and especially in the context of illness progression.
